# HIV seroprevalence, self-reported STIs and associated risk factors among men who have sex with men: a cross-sectional study in Rwanda, 2015

**DOI:** 10.1136/sextrans-2017-053311

**Published:** 2018-04-21

**Authors:** Roman Saba Ntale, Gad Rutayisire, Pierre Mujyarugamba, Eliah Shema, Jane Greatorex, Simon David William Frost, Pontiano Kaleebu

**Affiliations:** 1 Department of Biomedical Laboratory Sciences, College of Medicine and Health Sciences, University of Rwanda, Kigali, Rwanda; 2 Public Health England, Addenbrookes Hospital, University of Cambridge, Cambridge, UK; 3 Department of Veterinary Medicine, University of Cambridge, Cambridge, UK; 4 Uganda Virus Research Institute, Entebbe, Uganda

**Keywords:** hiv, infectious diseases, tropical stds, neisseria gonorrhoea, homosexuality

## Abstract

**Objectives:**

In many populations, men who have sex with men (MSM) are at a high risk of HIV infection. This study aimed to estimate the burden of HIV, other STIs and risk behaviours among Rwandan MSM.

**Methods:**

In this cross-sectional study, we recruited through peer referral men aged between 18 and 60 years, who reported sex with men at least once in the 12 months prior to the survey. Representativeness was increased using ‘seeds’ from a variety of sources. Signed informed consent was obtained from all participants. Data on demographics, risk behaviours and self-reported STIs were collected through an interviewer-administered questionnaire. We screened all eligible participants for HIV using the Rwanda-approved protocol for rapid HIV detection.

**Results:**

504 MSM were recruited from five major cities in Rwanda. Participants were mostly young (median age 23 years, range 18–55 years) and unmarried (484/504, 96.0%). Thirteen per cent (65/504) of the participants reported past gonorrhoea and/or syphilis infection. Of 504 MSM, 53 (10.5%) reported they were diagnosed and treated for gonorrhoea in the past 12 months and 24 (4.8%) tested positive for HIV. A high proportion (232/504, 46%) reported receiving payment for sex by a man, with almost half of these reporting on more than three occasions (107/232, 46%). Many reported having had an HIV test within the past 12 months (385/504, 76.4%). In multivariate logistic regression models controlling for age, being paid for sex was associated with higher odds of past STI (OR 3.36 (1.82–6.43]; P<0.001) and testing HIV positive (OR 3.13, P<0.05).

**Conclusion:**

Further research is needed to understand the high rate of payment for sex in this population, which appears to be a major risk factor for STI including HIV.

## Introduction

Men who have sex with men (MSM) are at a high risk of HIV infection.^1^ HIV-infected individuals have a considerably lower risk of developing AIDS or other serious illnesses if they start early antiretroviral treatment (ART).^2 3^ ART that results in low viral load (≤400 copies/mL) has been shown to reduce the risk of HIV transmission to uninfected sexual partners.^4 5^ While vulnerable groups like MSM would particularly benefit from treatment interventions, such control mechanisms may not be sought by these groups due to illegalisation, homophobia and stigmatisation.^1 6^ In order to address both the immediate risks and the underlying causes of vulnerability to HIV infection in limited resource settings, cost-effective control mechanisms require the determination and complete understanding of behavioural risks that drive the epidemic. As in many sub-Saharan countries, little is known about the prevalence of HIV, other STIs and risk behaviours among MSM in Rwanda.

The past research work of Chapman *et al* 7 reported a number of possible HIV risky behaviours such as frequent unprotected anal intercourse and multiple male sexual partnerships among a sample of 99 MSM living in Kigali City. Our study aimed to estimate the prevalence of HIV and other STIs, as well as characterising important risk behaviours among Rwandan MSM.

## Methods

Between January and March 2015, we conducted a cross-sectional biobehavioural study of active MSM in five Rwandan cities; Kigali, Rubavu, Muhanga, Musanze and Huye. Active MSM were defined as those reporting sex with a man (anal or oral sex) at least once in the 12 months prior to data collection. Active MSM, 18 years of age or older and living in any of the five Rwanda cities during the time of the study were anonymously recruited through snowball sampling strategies, which involved peer referrals with a double-incentive structure. Recruitment was targeted in cities as it was believed to be easier to recruit MSM into a research study than in rural areas.

To avoid stigmatisation of potential recruits and to encourage attendance, interviewers met participants at independent sites, preferably district hospital settings. These were organised in order to assure participants’ privacy; a waiting room and separate counselling and testing rooms were provided at each venue. Only those who presented themselves to the research venues were considered for recruitment. Multiple schedules were organised for each venue and for each visit. Individuals were encouraged to tell others to come for testing on the next scheduled visit. Anonymity was achieved through identification codes that were provided by trained enrolment officers. Diversity in the sample was increased by reaching a large number of initial contacts from a variety of sources, which included peer leaders, individuals who previously stayed in prison and middle-aged (40–60 years) participants. Participants were administered a questionnaire, which included questions on demographics, risk behaviours and self-reported STIs. For participants who could not read, the research team helped participants to complete the questionnaire through one-on-one oral communication. All subjects deemed active MSM living or working in any of the five Rwanda cities and 18 years of age or more were included if they were able to consent and willing to answer screening interview questions. All eligible participants were then screened for HIV by skilled technical personnel different from those involved in phlebotomy in order to shield the participant’s HIV infection status and using the approved national algorithm for HIV rapid testing.^8^Counselling was provided and all HIV-positive MSM were directed to MSM-friendly clinic.

Basic descriptive statistics were used to describe the cohort, overall and by HIV status, as well as by report of past STI. Logistic regression was used to determine factors associated with (a) HIV-positive serostatus and (b) reports of past STIs (gonorrhoea or syphilis) as estimates of ORs are typically robust to sampling strategy. Continuous variables (eg, age) were kept as continuous for analysis purposes. For multivariate analysis, we included variables that were significant at the P=0.1 level in univariate analyses using a likelihood ratio test between models with and without the variable. Due to the limited size of the dataset, only main effects (rather than interactions) were considered. Individuals with missing data for any of the variables in the analysis were excluded but this was only a small number of individuals (if any). Data were analysed using R V. 3.2 (Vienna, Austria).

## Results

In total, 504 MSM were recruited from five Rwandan cities; Kigali City (n=227), Ruhango (n=62), Huye (n=107), Musanze (n=44) and Rubavu (n=64). Participants were mostly young, with a median age of 23 years, (IQR 21–26) and unmarried (96.0%; 484/504). However, 97 of these men cohabited with a man. Sixty-three per cent (318/504) of the participants had multiple sex partners, with over a third of these (139/318) having more than three sex partners. The majority of participants practised anal sex with men (91%; 458/504), while about 11% (55/504) also practised vaginal sex with women. Also, >40% (207/504) reported inconsistent use of condoms and, together with those who reported never using condoms, >60% (306/504) of the participants reported unprotected sex.

Of the 65/504 (13%) participants who reported diagnosis with gonorrhoea and/or syphilis infection in the past year, 50 (10.0%) reported gonorrhoea in the past six months, while 12 (2.4%) had syphilis. Three participants reported both gonorrhoea and syphilis. A high proportion reported having had an HIV test (with negative results) in the year prior to the survey (385/504, 76.4%). Furthermore, 232/504 (46%) reported being paid for sex by a man, with almost half of these reporting on more than three occasions (107/232, 46%). Use of alcohol before sex by the partner or by the participant was reported by 74% and 69%, respectively. Use of drugs before sex in this population was reported by <10% (40/504).

In total, 24 of the 504 (4.8%) men tested positive for HIV. Individuals aged 23 or older (table 1) were more likely to test positive for HIV (OR 1.11, 95% CI 1.03 to 1.20; P=0.01). The prevalence of HIV seropositivity for the individual cities was 7.8%, 5/64; 4.8%, 3/62; 4.6%, 2/44; 4.4%, 10/227% and 3.7%, 4/107 for Rubavu, Ruhango, Musanze, Kigali and Huye, respectively. Of the 24 HIV-positive individuals, 3 reported also having sex with women. In multivariate logistic regression models, individuals being paid for sex were three times more likely to test positive for HIV (OR 3.13, 95% CI 1.30 to 8.43; P=0.015) and self-reporting on other STIs (OR 3.35, 95% CI 1.82 to 6.44; P<0.001).

**Table 1:** Univariate and multivariate logistic modelling of factors associated with STIs and HIV

Variable	STI (excluding HIV)				HIV			
	Univariate		Multivariate					
	OR	P values	OR	P values				
Age								
Per year	1.08 (1.02 to 1.14)	0.010	1.06 (0.10 to 1.13)	0.056	1.11 (1.03 to 1.20)	0.003	1.11 (1.03 to 1.20)	0.005
Education								
None/primary	1.0	0.105*			1.0	0.250*		
Secondary	0.60 (0.32 to 1.16)	0.116			0.49 (0.20 to 1.34)	0.133		
University	0.39 (0.15 to 0.94)	0.042			0.36 (0.08 to 1.34)	0.146		
Marital status								
Unmarried	1.0	0.611*			1.0	0.320*		
Cohabiting with man	1.50 (0.79 to 2.74)	0.199			2.23 (0.87 to 5.30)	0.077		
Single	1.27 (0.19 to 4.84)	0.762			1.91 (0.10 to 1.06)	0.546		
Married to woman†	1.90 (0.10 to 1.32)	0.570			N/A	N/A		
Number of sex partners								
1	1.0	0.072*	1.0		1.0	0.435*		
2	1.15 (0.51 to 2.49)	0.726	1.05 (0.45 to 2.38)	0.903	1.32 (0.33 to 4.73)	0.674		
3	0.93 (0.37 to 2.14)	0.861	0.60 (0.24 to 1.47)	0.282	1.5 (0.38 to 5.39)	0.539		
>3	2.12 (1.13 to 4.04)	0.020	1.30 (0.65 to 2.62)	0.459	2.33 (0.84 to 6.99)	0.111		
Timing of last sex act								
Within the last week	1.0	0.739*			1.0	0.147*		
<1 month	0.69 (0.36 to 1.27)	0.238			0.51 (0.18 to 1.26)	0.164		
1–3 months ago	0.72 (0.28 to 1.62)	0.448			0.21 (0.01 to 1.05)	0.131		
3–6 months ago	0.91 (0.30 to 2.32)	0.854			0.36 (0.02 to 1.87)	0.333		
6–12 months ago	1.25 (0.28 to 4.08)	0.740			N/A	N/A		
Type of sex								
Anal	2.25 (0.79 to 9.48)	0.186			N/A	N/A		
Vaginal	1.19 (0.53 to 2.45)	0.650			1.04 (0.24 to 3.13)	0.954		
Oral	1.26 (0.67 to 2.22)	0.472			0.89 (0.29 to 2.28)	0.826		
Masturbation	0.99 (0.46 to 1.96)	0.977			0.77 (0.18 to 2.30)	0.677		
Previous HIV test								
Yes (vs no)	1.04 (0.57 to 1.98)	0.913			0.50 (0.21 to 1.21)	0.107		
Current HIV status (excluded from multivariate)								
HIV+	4.54 (1.83 to 10.17)	0.0007			N/A	N/A		
Condom use								
Yes (vs no)	1.10 (0.58 to 2.23)	0.791			1.24 (0.46 to 4.33)	0.704		
Frequency of condom use								
Never	1.0	0.030*	1.0		1.0	0.077*		
Sometimes	1.53 (0.78 to 3.20)	0.238	1.38 (0.67 to 2.99)	0.400	1.86 (0.65 to 6.65)	0.284		
Every time	0.69 (0.32 to 1.53)	0.343	0.81 (0.36 to 1.87)	0.606	0.62 (0.16 to 2.55)	0.481		
Condom initiation								
None	1.0	0.33*			1.0	0.696*		
Partner	0.92 (0.32 to 2.42)	0.863			0.79 (0.11 to 4.16)	0.786		
Self	1.14 (0.6 to 2.35)	0.698			1.337 (0.485 to 4.713)	0.607		
Employed?								
Yes (vs no)	1.81 (1.07 to 3.1)	0.028	1.56 (0.89 to 2.76)	0.121	2.01 (0.88 to 4.74)	0.098	1.55 (0.66 to 3.73)	0.317
Monthly income								
<10 000	1.0	0.160*			1.0	0.190*		
10 000–50 000	1.43 (0.61 to 3.11)	0.383			2.40 (0.72 to 7.19)	0.129		
50 000–100 000	1.95 (1.06 to 3.56)	0.029			1.41 (0.47 to 4.01)	0.520		
>100 000	1.82 (0.64 to 4.50)	0.225			3.65 (0.95 to 11.93)	0.040		
Being paid for sex								
Yes (vs no)	3.90 (2.21 to 7.19)	5.13×10^–6^	3.36 (1.82 to 6.44)	0.0002	2.98 (1.26 to 7.83)	0.017	3.13 (1.20 to 8.43)	0.015
Frequency of being paid for sex (paid for sex used in multivariate instead)								
Never	1.0	1.23×10^–5^			1.0	0.123*		
Once per year	1.71 (0.47 to 4.93)	0.360			3.14 (0.66 to 1.87)	0.108		
Twice per year	4.82 (1.97 to 11.34)	0.000369			4.08 (1.03 to 4.17)	0.031		
Three times per year	3.23 (1.24 to 7.82)	0.011			1.75 (0.26 to 7.55)	0.492		
>3 times per year	4.80 (2.50 to 9.43)	3.28×10^–6^			3.05 (1.07 to 8.90)	0.036		
Alcohol use (self)								
Yes (vs no)	1.30 (0.73 to 2.39)	0.390			2.3 (0.85 to 7.99)	0.136		
Alcohol use (partner)								
Yes (vs no)	1.45 (0.79 to 2.87)	0.255			1.34 (0.53 to 4.10)	0.570		
Drug use (self)								
Yes (vs no)	1.02 (0.34 to 2.51)	0.964			1.81 (0.41 to 5.59)	0.354		
Drug use (partner)								
Yes (vs no)	0.96 (0.32 to 2.34)	0.934			1.71 (0.39 to 5.25)	0.404		
Inject drugs?								
Yes (vs no)	5.23 (1.01 to 24.24)	0.033	2.90 (0.50 to 15.41)	0.209	3.41 (0.18 to 21.15)	0.265		

*Overall p values.

†Men who have sex with men who are married to women.

N/A: Not applicable

## Discussion

To the best of our knowledge, this is the first paper reporting on the rates of HIV and other STIs in the MSM population in Rwanda. The prevalence of HIV within individual cities was almost twice as high in Rubavu, a busy border city close to the eastern part of the Democratic Republic of Congo. We also demonstrated a high prevalence of HIV (4.8%) compared with the Rwandan general population (3%),^9^ and to males of comparable age to our study population in the general population (0.5% in those aged 20–24 years). These findings are in agreement with the UNAIDS report (2010),^9^ showing that HIV prevalence is higher in MSM than in the general population. Nevertheless, HIV prevalence in our sample of Rwandan MSM is lower than in other parts of sub-Saharan Africa, where it can reach 17%–18%.^1^

Although individuals who engage in both vaginal and anal sex can act as a bridge for HIV in the general population,^10^ relatively few of our participants reported both. Despite this potential lack of bridging, the study participants belong to a high-risk group, with high rates of unprotected sexual intercourse and STI, as well as a high rate of HIV given the overall young demographics.

The inherent selection bias due to the snowball sampling methods may have been ameliorated as we included a variety of initial seeds in order to increase representativeness. Despite potential sampling biases, we recruited individuals belonging to a high-risk network. Unlike for HIV, STIs were self-reported and are likely to be underestimates of the true burden of STI. It is unavoidable that the participants may not provide honest responses to the sensitive questions covered in the questionnaire. However, this may have been ameliorated as all the research team members involved in data and sample collections were specifically trained to maintain the best relationship with the participants.

We have shown that MSM who received payments for sex are more likely to report STIs and be HIV-infected compared with those who do not. Further research is needed to help understand the high rate of payment for sex in this population. We suggest that HIV prevention interventions should in particular focus on factors driving STI risks in the youth and support facilities that endeavour to provide holistic health packages and/voluntary counselling and testing services, which allow inclusion of MSM as the country drives towards zero new HIV infections.
